# Using Behavioral Risk Factor Data as a surveillance tool to monitor the prevalence of initiation, continuation and completion of Human Papilloma Virus vaccination in children

**DOI:** 10.1016/j.dib.2016.03.005

**Published:** 2016-03-09

**Authors:** Gia Elise Barboza, Silvia Dominguez

**Affiliations:** Northeastern University, USA

**Keywords:** HPV, BRFSS, Sequential Logit, Andersen׳s Health Utilization

## Abstract

The Human Papilloma Virus (“HPV”) is a common sexually transmitted disease that has infected approximately 79 million men and women in the United States alone. A vaccination is available but in order to be effective it must be received prior to becoming sexually active and recipients must complete a three-dose sequence. In this article we explore the predisposing, enabling and need-based factors associated with parents’ or guardians’ decision to have their child initiate, continue and complete the Human Papilloma Virus (HPV) vaccine. The data file includes 5531 parents and guardians with presumptive knowledge regarding the number of HPV vaccination their child received. Data includes information on the child (e.g. child׳s age) as well as the adult respondent (e.g. health insurance status). A smaller subset of the dataset along with the code to run the model are supplied with this article. The interpretation of these data can be found in the research article published by the authors in the Journal of Preventive Medicine in 2015 http://dx.doi.org/10.1016/j.ypmed.2016.01.010[1].

**Specifications Table**

**Table:** 

Subject area	*Social Sciences*
More specific subject area	*HPV Vaccination*
Type of data	*Excel File*
How data was acquired	*A cross sectional landline and cellphone survey collected month with a standard questionnaire by the Centers for Disease Control and Prevention*
Data format	*Raw data*
Experimental factors	*Caregiver׳s initiation, continuation and completion of HPV vaccination among children ages 9–17 and categorization of health system beliefs*
Experimental features	*Data preparation; sequential logistic analysis to estimate the effect of predisposing, need-based and enabling factors on the odds of passing each transition*
Data source location	Connecticut, Kentucky, Pennsylvania, Texas, West Virginia and Wyoming (USA)
Data accessibility	*Data is included as a supplement to this article*

**Value of the Data**

• BRFSS data can be used to explore trends in the prevalence of HPV vaccination uptake and completion.
• BRFSS collects information on enabling, predisposing and need-based risk factors that can be used to explore HPV vaccination uptake and completion.
• BRFFS includes variables that can be used to explore the contextual aspects of HPV vaccination across smaller geographic units such as cities and counties.

## 1 Data

The data are from the Behavioral Risk Factor Surveillance System collected by the Centers for Disease Control in 2010 [2]. The BRFSS collects data on preventive health practices and risk behaviors that are linked to chronic diseases, injuries and infectious diseases that affect specific populations. In 2010, primary caretakers (e.g. parent or foster parent) of children between the ages of 9 and 17 from six states (Connecticut, Kentucky, Pennsylvania, Texas, West Virginia and Wyoming) were asked about HPV vaccination uptake and completion for one child living in their household. *A subset of the data along with the code to run the model is attached to this article so that the analysis can be reproduced. The larger dataset is publicly available from the CDC public repository.* http://www.cdc.gov/brfss/annual_data/annual_2014.html

## 2 Experimental design, materials and methods

### 2.1 Dependent variable

The dependent variable consisted of responses to the following two questions asked of BRFSS survey respondents: “A vaccine to prevent the human papilloma virus or HPV infection is available and is called the cervical cancer vaccine, HPV shot, or GARDASIL®. Has this child EVER had the HPV vaccination?” and “How many HPV shots did she receive?” The dependent variable was recoded in accordance with the assumptions of the sequential logistic regression model (see attached code for more details).

### 2.2 Independent variables

Anderson׳s Health Belief Model (HBM) [3], [4] was used to categorize those factors that may contribute to the vaccination decision: (1) enabling factors; (2) predisposing factors; and (3) need-based. Enabling factors included income (1=under $25,000 per year to 8=$75,000 or more), health insurance coverage such as HMOs or Medicare (1=yes, 0=no), access to at least one regular health care provider (1=yes, 0=no), affordability of care (1=yes, 0=no), routine checkup in past year (1=yes, 0=no) and place of residence (1=lives in a central city, 0=does not live in a central city). Predisposing factors included age and gender of both the primary caretaker (23–78) and the child (9–17), number of children residing in the household (1 to 5 or more), caregiver׳s highest level of educational attainment (1=less than High School to 6-post-baccalaureate), race/ethnicity (non-Hispanic white, non-Hispanic black, and Hispanic). The child׳s primary caretaker׳s general health status (1=fair/poor, 0=good/very good), number of days in either poor physical or mental health (1=2 or more weeks in past 30 days, 0=less than 2 weeks in past 30 days) and having receipt of a check-up in the past year (1=yes, 0=no) were identified as need-based.

### 2.3 Statistical analysis

The data were first analyzed using chi-square contingency analysis for bivariate data and then, in the main analysis, using a sequential logit regression [5] package that is available as an add-on in the STATA**®** statistical software package. The sequential logistic regression model was implemented to explore caregivers’ HPV vaccination decision for their child. We identified and conceptualized health related factors according to enabling, predisposing and need-based factors in accordance with Andersen׳s Health Belief Model. We downloaded the sequential logit package available in STATA using the findit command (findit seqlogit). Sequential logistic regression models is useful for modeling transitions through the health care system. In the present case, sequential logistic regression analysis estimated the effect of a set of explanatory variables on the odds of passing a specified number of transitions. At each transition, the model predicts the effect of the set of explanatory variables on the transition using the sample of persons who are ‘at risk’ of passing that transition (from 0 to 1 dose, from 1 to 2 doses, etc.). Adjustments were made to account for the BRFSS’ cluster design sampling scheme, which we incorporated into the analysis using a command option included in the sequential logit package.

Example code (hpv_code.txt) and data file (hpv_brfss10.csv) supplied with this Data in Brief includes a smaller subset of variables than in the published article. The data includes five variables included in the larger BRFSS data file: 1) our dependent variable that measures the number of HPV shots received by the child (0–3), child gender, child age (9–17), a dummy variable codifying whether the primary caregiver׳s race is white (1=white, 0=non-white) and whether the respondent caregiver has health insurance (1=yes, 0=no).
