# Supplementary material for: Using Behavioral Risk Factor Data as a surveillance tool to monitor the prevalence of initiation, continuation and completion of Human Papilloma Virus vaccination in children
Source: Data Brief. 2016 Mar 9;7:1614–6. doi: 10.1016/j.dib.2016.03.005 (PMC4866533; doi:10.1016/j.dib.2016.03.005)
Supplement: Supplementary file 2 — Supplementary material [file mmc2.docx]

In the present case there are three transitions that codify the effect of completing the first dose, the second dose, and all three doses. In other words, we have the following setup for the dependent variable:

0 vs 1,2,3 (at least one shot)

0,1 vs 2,3 (1 shot versus more than 1)

0,1,2 vs 3 (less than three shots versus completion of 3-dose sequence)

In the present case the levels of the dependent variable match the actual number of shots received. In other words, 0 shots is coded as 0, 1 shot is coded as 1, etc. So the first transition is specified as having received “No Shot” vs. “Having received at least one shot” (i.e. 0 vs. 1,2, 3), for example.

In STATA, after following the directions in the Data in Brief article to install the sequential logistic module, run the following code:

seqlogit c_hpvshots health_insurance c_gender white childage , or tree(0 : 1 2 3, 1: 2 3, 2:3)

The first variable after the “seqlogit” command is the dependent variable that was recoded to reflect each transition. Information about weighting or other selection criteria can be incorporated using optional commands (see the help file that is included with the sequential logistic package). The optional “or” tells STATA to provide odds ratios. The tree option is required because it tells STATA how many transitions to be modeled. In the present case, three transitions were modeled: having received the first dose, having received the second dose given that the first dose was received, and having completed all three doses.

Once the model is run, a number of post-estimation commands can be specified given the objectives of the study.

**Marginal effect of health_insurance

margins, dydx(health_insurance )

**Probability of achieving outcome 1

margins, dydx(health_insurance ) predict(pr outcome(1))

** Probability of choosing 1 in transition 1

margins, dydx(*) atmeans predict (trpr transition(1) choice(1))

** Explore a variables influence on each transition and the importance that a variable has on “passing” each transition (i.e. the weight of each transition as described in the help file for the sequential logit module).

seqlogitdecomp, table
